# Multimodal habitat radiomics based on automated breast volume scanning and conventional ultrasound for risk stratification of biopsy-confirmed BI-RADS 4A breast lesions

**DOI:** 10.3389/fonc.2026.1828818

**Published:** 2026-06-30

**Authors:** Fei Xia, Yu Zhang, Banghong Qiang, Junli Wang

**Affiliations:** Department of Ultrasound, WuHu Hospital, East China Normal University (The Second People’s Hospital, WuHu), Wuhu, Anhui, China

**Keywords:** automated breast volume scanning, BI-RADS 4A, breast lesions, habitat radiomics, ultrasound

## Abstract

**Background:**

To develop and validate a multimodal habitat radiomics model integrating automated breast volume scanning (ABVS) and conventional two-dimensional ultrasound (2D-US) for risk stratification of biopsy-selected BI-RADS 4A breast lesions.

**Methods:**

This retrospective single-center study included 160 consecutive patients with BI-RADS 4A breast lesions confirmed by histopathology between January 2024 and May 2025. Tumoral and peritumoral regions were manually segmented on ABVS and 2D-US images. Habitat subregions were generated using a local spatial autocorrelation-based heterogeneity analysis. Radiomic features were extracted using PyRadiomics. Feature selection was performed using t-test filtering, Pearson correlation analysis, and least absolute shrinkage and selection operator (LASSO) regression within the training folds. Multiple machine learning classifiers were constructed using three-fold cross-validation. Model performance was evaluated using area under the receiver operating characteristic curve (AUC), accuracy, sensitivity, specificity, F1 score, calibration curves, and decision curve analysis (DCA).

**Results:**

Of the 160 lesions, 51 (31.9%) were malignant and 109 (68.1%) were benign. The habitat radiomics model outperformed the clinical-ultrasound model. The optimal multilayer perceptron classifier achieved an AUC of 0.910 and an accuracy of 0.869 in the validation cohort. Decision curve analysis demonstrated higher net benefit across a range of threshold probabilities, and calibration curves indicated good agreement between predicted and observed outcomes.

**Conclusion:**

Multimodal habitat radiomics integrating ABVS and conventional ultrasound demonstrated promising performance for risk stratification of biopsy-selected BI-RADS 4A lesions and may provide supportive information for individualized clinical decision-making. Further prospective multicenter validation is warranted before clinical application.

## 1 Introduction

Breast cancer remains the most frequently diagnosed malignancy among women worldwide. Accurate risk assessment of suspicious breast lesions is essential for optimizing patient management and reducing overtreatment (1, 2).In clinical practice, BI-RADS 4A breast lesions fall within an indeterminate range for benign–malignant differentiation and are often regarded as a diagnostic “gray zone.” Although their reported risk of malignancy is relatively low (approximately 2%–10%), routine biopsy may lead to unnecessary psychological distress, increased healthcare costs, and procedure-related complications, thereby potentially affecting the appropriateness of clinical decision-making (3).Radiomics has developed rapidly in recent years. By extracting high-dimensional quantitative features from medical images, it provides a novel and objective approach for the assessment of breast lesions (4). Studies have demonstrated that multimodal radiomics can leverage complementary information across different imaging modalities, thereby enhancing model robustness and generalizability (5).Automated Breast Volume Scanning (ABVS), as a novel three-dimensional imaging technique, enables the acquisition of standardized volumetric breast images while reducing operator dependence and variability in image quality (4). Owing to its stable spatial resolution and abundant grayscale structural information, ABVS has emerged as an ideal data source for constructing radiomics models in breast imaging (6).

In oncologic imaging research, the concept of “habitat” is used to characterize intratumoral spatial heterogeneity by differentiating distinct microenvironmental subregions within a tumor, thereby enhancing the biological relevance of imaging features. Previous studies have demonstrated that habitat-derived features are closely associated with treatment response, prognosis, and underlying molecular characteristics (7).For example, Shi et al. (8) developed a habitat-based radiomics framework derived from dynamic contrast-enhanced MRI (DCE-MRI) to characterize the tumor microenvironment and predict response to neoadjuvant chemotherapy in breast cancer, further supporting the value of habitat radiomics in quantifying intratumoral heterogeneity. However, to date, studies applying habitat radiomics based on multimodal imaging—specifically two-dimensional gray-scale ultrasound combined with ABVS—for BI-RADS 4A breast lesions remain limited. Based on this rationale, the present study applied a habitat radiomics approach to extract quantitative features from two-dimensional gray-scale ultrasound and ABVS images, and constructed a multimodal habitat radiomics model. Its performance was compared with that of a machine learning model based on clinical and conventional ultrasound features. The aim was to evaluate the potential value of this approach for risk stratification and optimization of clinical management strategies in BI-RADS 4A breast lesions.

## 2 Materials and methods

### 2.1 Study population

This retrospective study was approved by the institutional review board, and informed consent was waived. Ethical approval number: 2024-KY-103. We included consecutive patients diagnosed as BI-RADS 4A who subsequently underwent biopsy between January 2024 and May 2025. Only lesions with definitive histopathological results were included. This design represents a biopsy-selected cohort rather than a screening population. Inclusion criteria:①BI-RADS 4A assessment before biopsy;②Availability of both ABVS and 2D-US images;③Complete clinicopathological data. Exclusion criteria:①Prior neoadjuvant therapy;②Poor image quality.

### 2.2 Equipment and imaging protocols

Ultrasound examinations were performed using a Siemens Acuson S2000 system (Siemens Medical Solutions, Mountain View, CA, USA) equipped with a 14L5 linear array transducer (7–14 MHz) and a 14L5BV automated volume transducer (5–14 MHz). Acoustic radiation force impulse (ARFI) imaging and automated breast volume scanning (ABVS) functions were enabled during the examinations. Patients were positioned in the supine position with both arms naturally raised or placed above the head to ensure full exposure of the breasts, while maintaining steady breathing. A radiologist who had received specialized training in ABVS first performed conventional two-dimensional gray-scale and color Doppler ultrasound examinations, documenting lesion characteristics including gray-scale features and vascular flow patterns. Subsequently, acoustic radiation force impulse (ARFI) imaging was performed in the optimal imaging plane, including both qualitative elastography and quantitative modes, to measure the elasticity score and shear wave velocity (SWV) of the lesion. During ABVS acquisition, appropriate preset parameters were selected. The probe was gently placed on the breast surface with slight compression to stabilize the breast tissue. Whole-breast scanning was performed according to the standardized protocol, including anteroposterior, lateral, and medial views. After image acquisition, all datasets were transferred to the workstation for post-processing. Three-dimensional reconstruction was performed using the ABVS system to generate coronal, transverse, and sagittal images for subsequent analysis.

### 2.3 Ultrasound image analysis

Image analysis was independently performed in a double-blinded manner by two radiologists who had completed specialized training in ABVS. In cases of disagreement, a consensus was reached through joint review. The following variables were recorded: patient age; maximum lesion diameter; lesion location and quadrant; margin characteristics; orientation; internal echogenic pattern; presence of microcalcifications; Adler vascularity grade; elasticity score; shear wave velocity (SWV); and the presence of the retraction phenomenon on the coronal plane.

### 2.4 Tumor segmentation and habitat subregion generation

After exporting the two-dimensional gray-scale ultrasound and ABVS images, lesion segmentation was performed using 3D Slicer software (version 5.2.2). One radiologist with 5 years of experience in ultrasound diagnosis manually delineated the target lesion and peritumoral region, while another senior radiologist with 10 years of experience reviewed the segmentation results. To generate subregions within the regions of interest, a method based on local spatial autocorrelation and regional attribute values was employed to identify spatial patterns with significant heterogeneity within the lesion. The specific workflow was as follows:

First, the input three-dimensional medical imaging data and corresponding mask files were loaded, and the regions of interest were defined. The input images provided spatial attribute values, whereas the mask files labeled different regions. These input data were used to analyze the local spatial characteristics within different masked regions.

The corresponding voxel set within each mask was extracted, and the spatial coordinates and attribute values of the voxels were recorded. Assuming that the voxel set within a region is represented as {*x_i_*|*I* = 1,…,*n*}, where *x_i_* denotes the attribute value of each voxel and *n* represents the total number of voxels within the region, the mean attribute value $\bar{x}$ of the region was calculated as a global statistical descriptor. The overall variance $M_{2}=\frac{1}{n}\sum_{i=1}^{n}{\left(x_{i}-\bar{x}\right)}^{2}$ within the region was also computed as a normalization factor.

To capture local spatial correlations among voxels within the region, a neighborhood-based spatial weight matrix *W* was constructed. The weight matrix was determined according to the Euclidean distance between voxels. Specifically, if the distance between two voxels was less than or equal to the diagonal distance of the eight-neighborhood connection $\sqrt{3}$, the voxels were defined as neighbors and assigned a weight *w_ij_* = 1; otherwise, *w_ij_* = 0. Based on these definitions, the spatial weight matrix *W*formed a symmetric sparse matrix representing local voxel spatial relationships within the region.

For each voxel *i* within the region, the local Moran’s I statistic was calculated. The local Moran’s I was defined as:


$$I_{i}=\frac{\left(x_{i}-\bar{x}\right)}{M_{2}}\sum_{j}w_{ij}(x_{j}-\bar{x}),$$

where *x_i_* and *x_j_* represent the attribute values of voxel *i* and its neighboring voxels, respectively; $\bar{x}$ is the mean attribute value of the region; *M*_2_ is the regional variance; and *w_ij_* is derived from the spatial weight matrix *W*. The local Moran’s *I_i_* value describes the spatial association between a voxel *i* and its neighboring voxels. Positive *I_i_* values indicate that the voxel and its neighboring voxels exhibit similar attribute distributions, representing positive spatial autocorrelation, whereas negative *I_i_* values indicate substantial differences between the voxel and its neighboring voxels, representing negative spatial autocorrelation.

Based on the local Moran’s I values and voxel attribute values, each voxel was reclassified to generate a new habitat mask. The classification strategy was as follows: first, the absolute value of *I_i_* was compared with a predefined significance threshold. Voxels with *I_i_* below the threshold were categorized as non-significant regions. Otherwise, classification was further performed according to the sign of *I_i_*. For *I_i_* > 0, representing positive spatial autocorrelation, voxels were classified as High-High (HH) or Low-Low (LL) regions according to the relationship between voxel intensity *x_i_* and the regional mean $\bar{x}$. For *I_i_* < 0, representing negative spatial autocorrelation, voxels were classified as High-Low (HL) or Low-High (LH) regions according to the relationship between *x_i_* and $\bar{x}$. Ultimately, a new habitat mask was generated in which each voxel was assigned to one of five categories: High-High, Low-Low, High-Low, Low-High, or non-significant regions.

**Figure 1:**
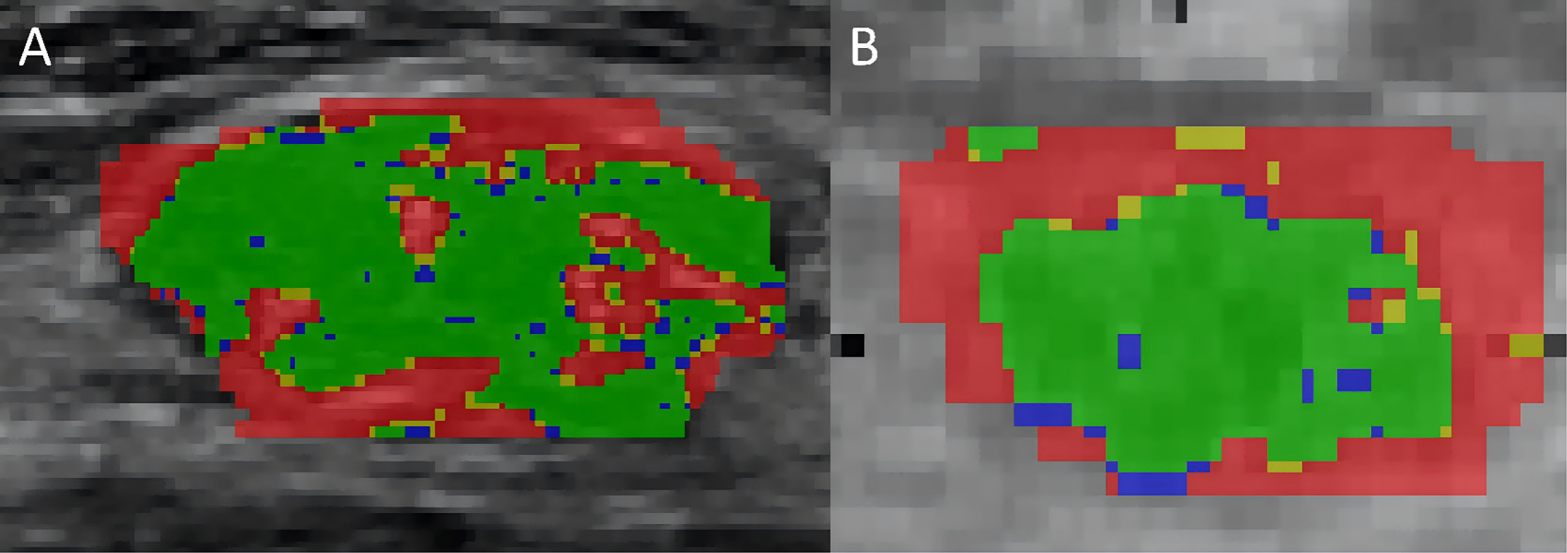
Representative visual examples of habitat subregion overlays on ultrasound images. **(A)** Habitat subregion overlay generated from conventional two-dimensional gray-scale ultrasound (2D-US) images. **(B)** Habitat subregion overlay generated from automated breast volume scanning (ABVS) images.Colors are displayed for visualization purposes only.

After habitat classification of all regions of interest was completed, all subregions were merged into a global mask while preserving spatial coordinate information and habitat labels. The generated masks were subsequently saved in standard NIfTI format for subsequent radiomics feature extraction from the significant habitat regions. By combining local spatial autocorrelation analysis with voxel attribute classification, this method effectively identified spatial heterogeneity within larger regions and enabled extraction of habitat-level radiomics features. The above habitat subregion generation process was implemented using the PixelMed AI platform. Representative visual examples of habitat subregion overlays on ultrasound images are shown in **Figure 1**.

### 2.5 Radiomics feature extraction

Radiomic features were extracted separately from the intratumoral and peritumoral habitat subregions. Prior to feature extraction, ultrasound images underwent preprocessing. All images and corresponding segmentation masks were resampled to a uniform pixel spacing of 2 × 2 mm², followed by gray-level discretization. In our dataset, ultrasound images were acquired using a standardized protocol but still exhibited minor inter-image variability in pixel spacing. Resampling to 2 × 2 mm² ensured that all radiomic features were extracted under identical spatial conditions, which is a prerequisite for stable and reproducible radiomics pipelines. Radiomics features were extracted from both original and filtered images using PyRadiomics software, including wavelet-transformed and Laplacian of Gaussian (LoG)-filtered features. The extracted features from the training and validation sets were subsequently standardized using Z-score normalization.

### 2.6 Feature selection and model development

To identify radiomic features with good reproducibility and low redundancy, a multi-step feature selection strategy was applied. First, independent sample t-tests were performed on features derived from different habitat subregions, and features without statistically significant differences (P > 0.05) were excluded. Subsequently, Pearson correlation coefficients were calculated to assess feature redundancy. For feature pairs with a correlation coefficient greater than 0.9, only one feature was retained. Further dimensionality reduction was conducted using least absolute shrinkage and selection operator (LASSO) regression. The optimal regularization parameter (λ) was determined via ten-fold cross-validation, and features with non-zero coefficients were selected for model construction. Finally, a bar plot of the selected features and their corresponding LASSO coefficients was generated to evaluate feature importance.

**Figure 2:**
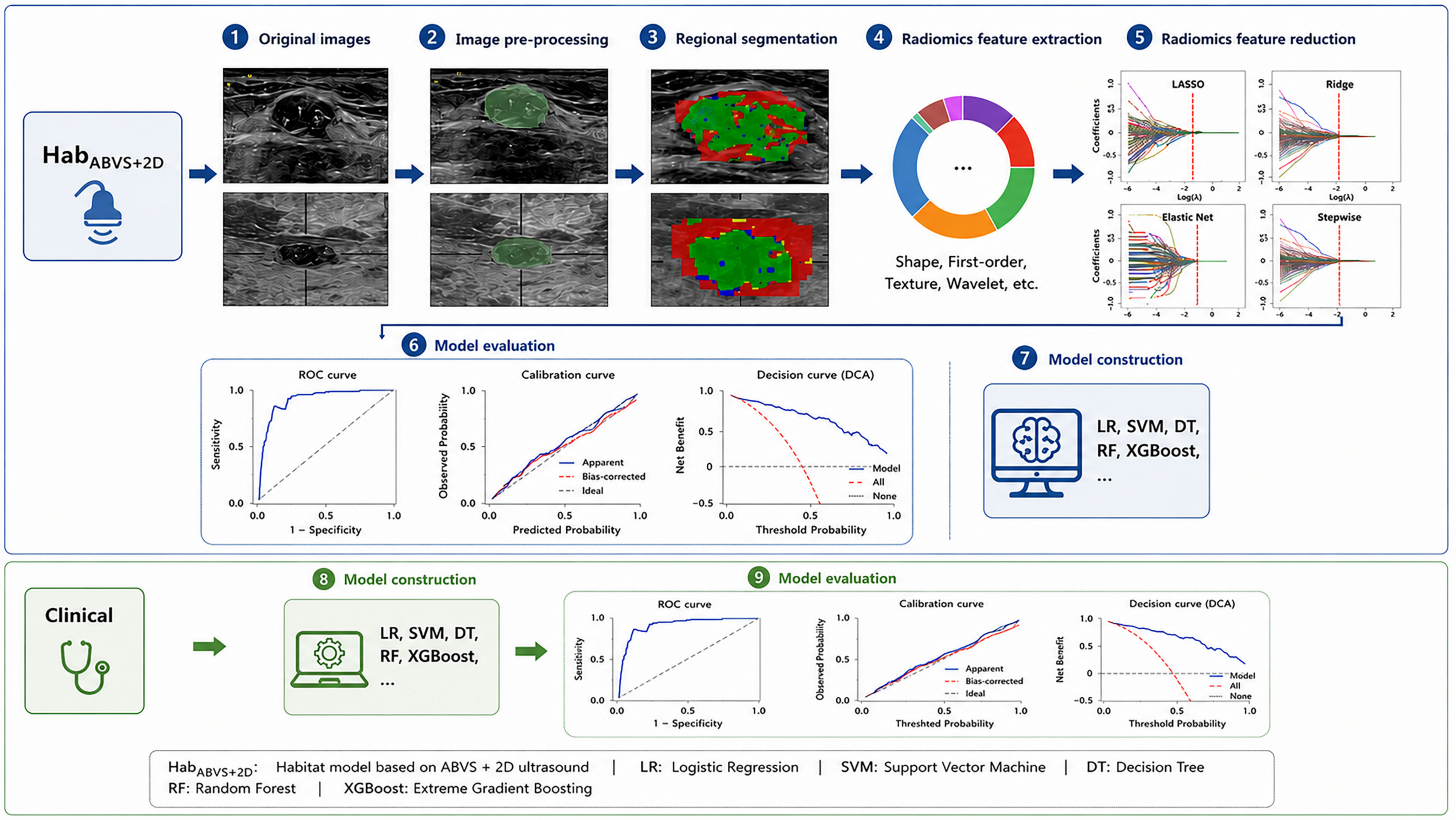
Workflow diagram. This image illustrates the segmentation, feature extraction, and feature selection processes of ABVS and two-dimensional grayscale ultrasound images for BI-RADS 4A breast lesions. ABVS, Automated Breast Volume Scanner; HabABVS+2D, habitat radiomics model based on ABVS combined with two-dimensional grayscale ultrasound.

After feature fusion and selection, two models were established: an intratumoral habitat radiomics model and a clinical model based on conventional clinical and ultrasound features. Machine learning classifiers were constructed using standard machine learning libraries. The overall workflow of model development is illustrated in **Figure 2**. The following classifiers were evaluated: Logistic Regression (LR), Support Vector Machine (SVM), Decision Tree (DT), Random Forest (RF), Extremely Randomized Trees (ExtraTrees), eXtreme Gradient Boosting (XGBoost), Multi-Layer Perceptron (MLP), Gradient Boosting Machine (GBM), and Light Gradient Boosting Machine (LightGBM). Three-fold cross-validation was performed to assess model stability and generalization performance. Receiver operating characteristic (ROC) curves were generated, and the area under the curve (AUC) was calculated to evaluate diagnostic performance. To avoid data leakage, all preprocessing procedures, including feature normalization, statistical filtering, correlation analysis, feature selection, and model training, were performed exclusively within each training fold during cross-validation, while the corresponding validation fold remained completely independent for performance evaluation.

### 2.7 Statistical analysis

Statistical analyses were performed using Python software (version 3.8.2). Quantitative variables with normal distribution were compared using the independent-samples t-test and are presented as mean ± standard deviation. Non-normally distributed variables were analyzed using the Mann–Whitney U test and are expressed as median (interquartile range). Categorical variables were compared using the chi-square (χ²) test and are presented as counts (percentages).

All statistical tests were two-sided, and a P value < 0.05 was considered statistically significant. Model performance was evaluated using receiver operating characteristic (ROC) curves and the corresponding area under the curve (AUC). Decision curve analysis (DCA) was performed to assess the clinical utility of the models.

## 3 Results

### 3.1 Development of the clinical model

A total of 160 female patients with 160 BI-RADS 4A breast lesions were included, comprising 51 malignant and 109 benign lesions.

The clinical model incorporated the following variables: age; maximum lesion diameter; quadrant location; laterality; lesion orientation; margin characteristics; shape; internal echogenic pattern; presence of microcalcifications; Adler vascularity grade; elasticity score; shear wave velocity (SWV); and the presence of the retraction phenomenon on the coronal plane.

Based on these clinical and conventional ultrasound features, multiple machine learning algorithms were applied to construct predictive models. The mean area under the curve (AUC) and accuracy (ACC) from three-fold cross-validation are presented in **Table 1**. Among the evaluated classifiers, the Gradient Boosting Machine (GBM) demonstrated relatively superior performance in both the training and validation sets, achieving AUCs of 0.880 and 0.707, and ACCs of 0.848 and 0.725, respectively.

**Table 1:** Performance comparison of clinical models constructed using different machine learning algorithms based on three-fold cross-validation.

Model	AUC	ACC	Task
LR	0.793	0.791	Training set
LR	0.758	0.794	Validation set
SVM	0.783	0.784	Training set
SVM	0.753	0.794	Validation set
DT	0.817	0.781	Training set
DT	0.701	0.694	Validation set
RF	0.846	0.793	Training set
RF	0.682	0.763	Validation set
ExtraTree	0.817	0.753	Training set
ExtraTree	0.697	0.707	Validation set
XGBoost	0.865	0.763	Training set
XGBoost	0.695	0.744	Validation set
MLP	0.226	0.472	Training set
MLP	0.229	0.451	Validation set
GBM	0.880	0.848	Training set
GBM	0.707	0.725	Validation set
LightGBM	0.783	0.725	Training set
LightGBM	0.648	0.619	Validation set

### 3.2 Selection of habitat radiomic features

**Figure 3:**
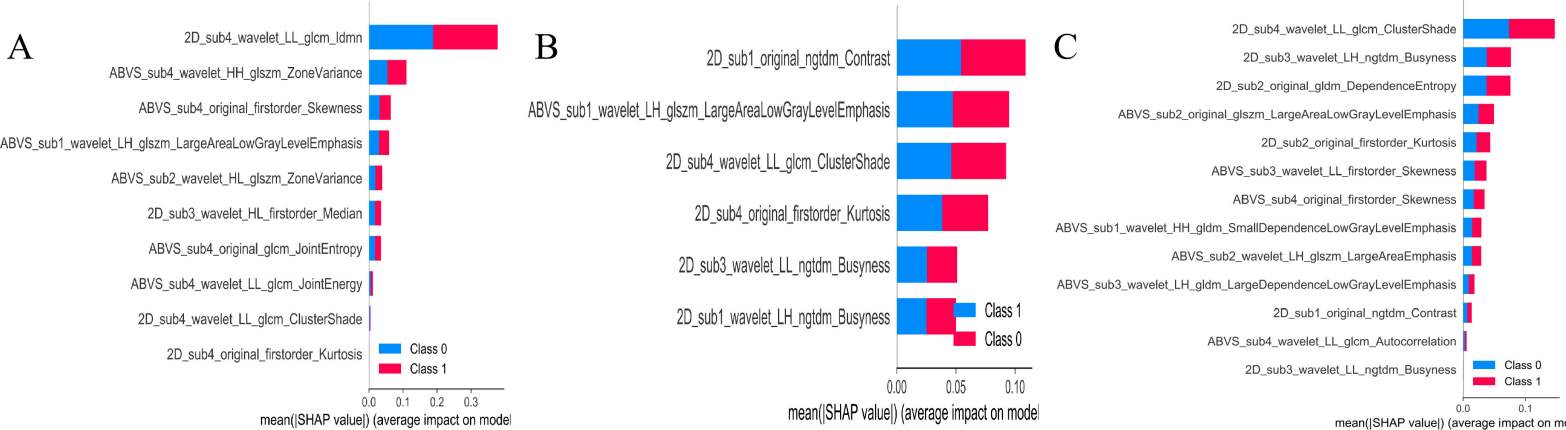
SHAP importance ranking plot of habitat radiomics features. The horizontal axis represents the mean absolute SHAP value, indicating the contribution of each feature to the model’s predictions; the vertical axis lists the radiomics feature names. Different colors denote the direction of each feature’s impact on the prediction of benign and malignant outcomes. **(A)** SHAP plot for fold 1; **(B)** SHAP plot for fold 2; **(C)** SHAP plot for fold 3.

A total of 3,715 radiomic features were extracted from the ABVS and two-dimensional gray-scale ultrasound images of BI-RADS 4A breast lesions. After preprocessing and dimensionality reduction using LASSO regression, 10, 6, and 13 features were selected in the three respective cross-validation folds for model construction. The SHAP-based importance ranking of the selected features in each fold is presented in **Figure 3**.

### 3.3 Development of the habitat radiomics model

Based on radiomic features extracted from ABVS and two-dimensional gray-scale ultrasound images, multiple machine learning algorithms were used to construct the habitat radiomics models. The mean AUC and accuracy (ACC) obtained from three-fold cross-validation are summarized in **Table 2**. Among the evaluated classifiers, the multilayer perceptron (MLP) achieved the best diagnostic performance in both the training and validation sets, with AUCs of 0.939 and 0.910, and ACCs of 0.897 and 0.869, respectively.

**Table 2:** Performance comparison of habitat radiomics models constructed using different machine learning algorithms based on three-fold cross-validation.

Model	AUC	ACC	Task
LR	0.792	0.756	Training set
LR	0.683	0.657	Validation set
SVM	0.751	0.744	Training set
SVM	0.705	0.632	Validation set
DT	0.807	0.790	Training set
DT	0.697	0.720	Validation set
RF	0.845	0.806	Training set
RF	0.804	0.812	Validation set
ExtraTree	0.772	0.741	Training set
ExtraTree	0.632	0.687	Validation set
XGBoost	0.942	0.922	Training set
XGBoost	0.883	0.845	Validation set
MLP	0.939	0.897	Training set
MLP	0.910	0.869	Validation set
GBM	0.893	0.847	Training set
GBM	0.825	0.820	Validation set
LightGBM	0.862	0.806	Training set
LightGBM	0.784	0.732	Validation set

### 3.4 Comparative evaluation of different models

**Figure 4:**
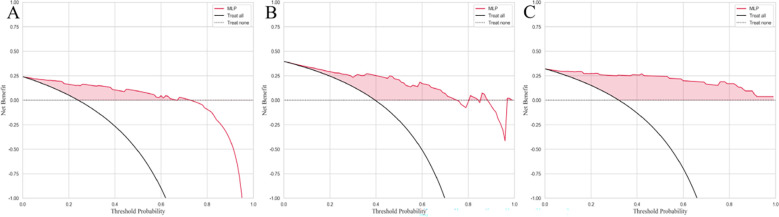
Decision curve analysis (DCA) results of the habitat radiomics model across different folds in the validation set. **(A)** DCA curve of the first fold (fold 1) in the validation set; **(B)** DCA curve of the second fold (fold 2) in the validation set; **(C)** DCA curve of the third fold (fold 3) in the validation set.

**Figure 5:**
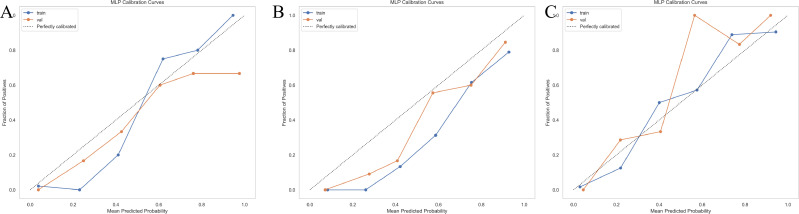
Calibration curves of the habitat radiomics model across different folds in the training and validation sets. **(A)** Calibration curve of the first fold (fold 1); **(B)** Calibration curve of the second fold (fold 2); **(C)** Calibration curve of the third fold (fold 3).

The cross-validation performance comparison between the best-performing clinical model (GBM) and the optimal habitat radiomics model (MLP) is presented in **Table 3**.The AUC values and corresponding 95% confidence intervals of the clinical-ultrasound model and habitat radiomics model across three-fold cross-validation are presented in **Table 4**.The results indicate that, in both the training and validation sets, the habitat radiomics model outperformed the clinical model in terms of AUC, accuracy (ACC), sensitivity, specificity, and F1 score. Decision curve analysis (DCA) was performed in the validation set (**Figure 4**) to evaluate the net clinical benefit of the models across a range of threshold probabilities. In addition, calibration curves were plotted for both the training and validation sets (**Figure 5**) to assess the agreement between predicted probabilities and actual outcomes.

**Table 3:** Cross-validation performance evaluation of the clinical model and the habitat radiomics model.

Model	AUC	ACC	SEN	SPE	NPV	PPV	F1
Training set							
Clinical model	0.880	0.848	0.757	0.870	0.886	0.766	0.751
Habitat radiomics model	0.939	0.897	0.950	0.876	0.974	0.794	0.856
Validation set							
Clinical model	0.707	0.725	0.627	0.769	0.813	0.556	0.588
Habitat radiomics model	0.910	0.869	0.919	0.842	0.959	0.751	0.817

**Table 4:** AUC values with corresponding 95% confidence intervals for the clinical-ultrasound model and habitat radiomics model across three-fold cross-validation.

Model	Fold1AUC (95% CI)	Fold2AUC (95% CI)	Fold3AUC (95% CI)	Task
Clinical model	0.892	0.891	0.856	Training set
	(0.831-0.948)	(0.819-0.944)	(0.780-0.909)	
	0.685	0.739	0.697	Validation set
	(0.547-0.834)	(0.620-0.869)	(0.556-0.859)	
Habitat radiomics model	0.982	0.909	0.925	Training set
	(0.951-1.000)	(0.858-0.955)	(0.902-0.989)	
	0.899	0.887	0.943	Validation set
	(0.824-0.964)	(0.820-0.961)	(0.934-0.995)	

## 4 Discussion

This study focused on BI-RADS 4A breast lesions, which pose substantial diagnostic challenges in clinical practice. We developed a machine learning–based clinical model incorporating conventional clinical and ultrasound features, as well as a multimodal habitat radiomics model integrating ABVS and two-dimensional gray-scale ultrasound. The diagnostic performance of the two models was systematically compared. The multimodal habitat radiomics model demonstrated superior performance over the clinical model, indicating its potential value for risk stratification of BI-RADS 4A breast lesions.

BI-RADS 4A breast lesions are characterized by a relatively low but non-negligible risk of malignancy, with prior studies reporting malignancy rates of approximately 2–10% (9). In routine clinical practice, however, most BI-RADS 4A lesions undergo biopsy to minimize the risk of missed cancer. As a result, the majority are ultimately confirmed as benign, leading to unnecessary invasive procedures, increased healthcare resource utilization, and psychological burden for patients (10). Therefore, how to provide supportive information for biopsy decision-making and improve clinical management has become one of the important directions in current breast imaging research. In our cohort, the malignancy prevalence (31.9%) was higher than the commonly reported 2–10% for BI-RADS 4A lesions. This discrepancy reflects the retrospective inclusion of biopsy-confirmed cases, representing a clinically selected higher-risk subgroup rather than a screening population.

Previous studies have explored radiomics or deep learning approaches for the risk stratification of BI-RADS 4A breast lesions using ABVS or conventional ultrasound imaging. Wang et al. (3) and Ma et al. (6) developed radiomics-based models derived primarily from single-modality imaging and whole-lesion region-of-interest (ROI) analysis, demonstrating improved diagnostic performance compared with conventional assessment. However, these approaches generally treated the lesion as a relatively homogeneous imaging unit and did not explicitly characterize intratumoral spatial heterogeneity or the potential contribution of distinct peritumoral microenvironmental subregions.

Conventional ultrasound primarily relies on morphological assessment and experience-based criteria. Although it can aid clinical decision-making to some extent, it is inherently subjective and limited in its ability to characterize intratumoral microstructural heterogeneity (11). In the present study, the model constructed solely from clinical and conventional two-dimensional gray-scale ultrasound features demonstrated limited generalizability in the validation set. In contrast, the habitat radiomics model, by incorporating high-dimensional quantitative features, significantly improved diagnostic performance for BI-RADS 4A breast lesions. These findings are consistent with previous studies suggesting that radiomics-based approaches may help reduce unnecessary biopsies (3, 12).

The principal advantage of habitat radiomics lies in its ability to move beyond conventional whole–region of interest (ROI) analysis by identifying and quantitatively characterizing distinct subregions within the tumor and peritumoral microenvironment that may exhibit different biological properties, thereby providing a more comprehensive representation of spatial heterogeneity (13, 14). Accumulating evidence has demonstrated that such intratumoral spatial heterogeneity is closely associated with tumor proliferation, invasiveness, metastatic potential, and adverse prognosis, representing a major challenge in precision oncology (15). Compared with conventional whole-ROI radiomics approaches, the habitat radiomics strategy used in the present study aimed to further characterize spatial heterogeneity by partitioning intratumoral and peritumoral regions into multiple habitat subregions based on local spatial autocorrelation patterns. Rather than extracting features from the lesion as a single entity, this framework enabled separate quantitative characterization of different spatially heterogeneous subregions that may reflect distinct biological properties. In theory, such an approach may provide additional information beyond conventional ROI-level analysis and may partially explain the improved performance observed in the present study.

The SHAP-based interpretability analysis further supported these findings. Radiomic features derived from different habitat subregions exhibited distinct contribution directions and relative importance in model prediction, indicating that various intratumoral microenvironmental subregions are not equivalent in distinguishing benign from malignant lesions. These results are consistent with prior MRI-based habitat radiomics studies in breast cancer conducted by Shi et al. (8) and Chen et al. (14), which highlighted the critical role of spatial heterogeneity in imaging-based predictive models. For BI-RADS 4A lesions—where the probability of malignancy lies in an intermediate and diagnostically challenging range—the incorporation of habitat-level information may provide meaningful clinical value in supporting risk stratification and decision-making.

According to Lambin et al., ABVS, as a standardized three-dimensional imaging technique with reduced operator dependence, provides a stable and reproducible data foundation for radiomics analysis (16). In the present study, ABVS-derived three-dimensional structural information was combined with conventional two-dimensional grayscale ultrasound to construct a multimodal habitat radiomics model. Compared with previously reported single-modality or conventional non-habitat radiomics approaches (3, 13, 17), the proposed framework achieved favorable diagnostic performance in differentiating benign from malignant BI-RADS 4A lesions within the current dataset. These findings suggest that integrating complementary imaging information together with spatial heterogeneity analysis may provide additional value for lesion characterization (18). However, given the retrospective design and lack of external validation, the potential incremental benefit of the proposed multimodal habitat framework should be interpreted cautiously and requires further confirmation in larger multicenter studies.

Decision curve analysis (DCA) showed that the habitat radiomics model achieved higher net benefit across a range of threshold probabilities. These findings suggest that the proposed model may provide supportive information for clinical risk assessment and biopsy decision-making. However, its actual clinical utility remains to be validated in prospective real-world settings.

This study has several limitations. First, it was a single-center retrospective study with a relatively limited sample size, In addition, the malignancy prevalence in our cohort (31.9%) was higher than that typically reported for BI-RADS 4A lesions because only biopsy-confirmed cases from a retrospective clinical cohort were included. Therefore, the study population represents a biopsy-selected high-risk subgroup rather than a general screening population, which may introduce selection bias and limit the generalizability of the model. Second, all imaging data were acquired using the same device, and the generalizability of the model still requires further validation across multiple centers and imaging platforms. In addition, although manual segmentation was performed and reviewed by experienced radiologists, formal inter-observer and intra-observer reproducibility analyses using intraclass correlation coefficients (ICC) were not conducted. Future studies should incorporate repeated segmentation and robustness analysis to further validate the stability of the extracted radiomics features.

In conclusion, The multimodal habitat radiomics model based on ABVS combined with two-dimensional gray-scale ultrasound demonstrated promising diagnostic performance for differentiating benign and malignant BI-RADS 4A breast lesions. However, given the retrospective single-center design and lack of external validation, further large-scale prospective multicenter studies are required before potential clinical application can be considered. The present study should be regarded as a proof-of-concept investigation. To develop a clinically applicable final model, retraining on substantially larger multicenter datasets using fixed and consensus-based feature sets will be necessary. Further validation across multiple centers, larger populations, and different imaging platforms is still required to confirm the stability and generalizability of the model and to facilitate its future clinical translation.

## Data Availability

The original contributions presented in the study are included in the article/supplementary material. Further inquiries can be directed to the corresponding author.
